# Genomic alteration profile and PD‐L1 expression among different breast cancer subtypes in Chinese population and their correlations

**DOI:** 10.1002/cam4.5314

**Published:** 2022-11-20

**Authors:** Kai Li, Li Cao, Cheukfai Li, Jundong Wu, Bo Chen, Guochun Zhang, Xueri Li, Lingzhu Wen, Minghan Jia, Guangnan Wei, Jiali Lin, Yingzi Li, Yuchen Zhang, Hsiaopei Mok, Chongyang Ren, Yulei Wang, Xiaofang Qi, Lijie Guo, Yue Che, Ning Liao

**Affiliations:** ^1^ Department of Breast Cancer, Guangdong Provincial People's Hospital Guangdong Academy of Medical Sciences Guangzhou China; ^2^ Guangdong Provincial Key Laboratory for Breast Cancer Diagnosis and Treatment Cancer Hospital of Shantou University Medical College Shantou China; ^3^ School of Medicine South China University of Technology Guangzhou China; ^4^ The Second School of Clinical Medicine Southern Medical University Guangzhou China; ^5^ OrigiMed Shanghai China

**Keywords:** breast cancer, Ki67, next‐generation sequencing, PD‐L1, TMB

## Abstract

**Backgroud:**

There were limitations existing in programmed cell‐death ligand 1 (PD‐L1) as predictive biomarkers for breast cancer (BC), hence exploring the correlation between PD‐L1 levels and other biomarkers in BC may become a very useful therapeutic clinical tool.

**Methods:**

A total of 301 Chinese patients with different BC subtypes including 47 HR+/HER2+, 185 HR+/HER2−, 38 HR−/HER2+, and 31 triple‐negative breast cancer (TNBC) were enrolled in our study. Next‐generation sequencing based Yuansu450 gene panel was used for genomic alteration identification and PD‐L1 expression was tested using immunohistochemistry.

**Results:**

The most prevalent BC‐related mutations were *TP53* mutations, followed by mutations in *PIK3CA*, *ERBB2*, *CDK12*, and *GATA3* in our Chinese cohort. We found that mutations *DDR2* and *MYCL* were only mutated in HR−/HER2+ subtype, whereas *H3‐3A* and *NRAS* mutations were only occurred in HR−/HER2− subtype. The percentage of patients with PD‐L1‐positive expression was higher in patients with HR‐/HER2− mainly due to the percentage of PD‐L1‐high level. Mutational frequencies of *TP53*, *MYC*, *FAT4*, *PBRM1*, *PREX2* were observed to have significant differences among patients with different BC subtypes based on PD‐L1 levels. Moreover, a positive correlation was observed between TMB and PD‐L1 level in HR+/HER2− subtype, and showed that the proportion of patients with high PD‐L1 expression was higher than that of patients with low PD‐L1 expression in the HR+/HER2− and HR+/HER2+ cohorts with high Ki67 expression.

**Conclusions:**

The genomic alterations based on PD‐L1 and other biomarkers of different cohorts may provide more possibilities for the treatment of BC with different subtypes.

## Graphical abstract

The mutational alterations based on PD‐L1 levels and the specific mutated genes in the different BC subtype were analyzed. Molecular testing will allow better understanding of disease biology and tailor therapy for specific individual. Verification of using integrated predictive biomarkers for selecting checkpoint inhibitors as a component of combination systemic therapy in BC is ongoing.

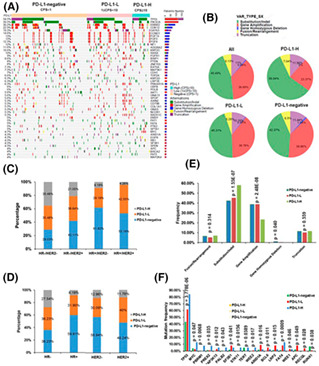

## 1 INTRODUCTION

Breast cancer (BC) is one of the leading causes of cancer death and a leading cause of morbidity and mortality among women worldwide.^1^ Immune checkpoint inhibitors (ICI) is becoming a new treatment mode in advanced BC. The discovery of selective ICI has created new opportunities for treatment and also aroused great interest in the use of immunotherapy on BC treatment.^2^ ICI therapies targeting PD‐1/PD‐L1 were observed to be useful against cancers at different sites, and numerous blocking antibodies are currently used in BC.^3^ Previous research have exhibited the diversity of PD‐L1 expression in different subtypes of BC, and the different responses of different BC subtypes to PD‐L1 inhibitors. For example, the IMPASSION 130 study showed improved PFS in PD‐L1‐positive triple‐negative BC (TNBC) subgroup treating with atezolizumab plus nab‐paclitaxel.^4^ The KEYNOTE‐355 trial results demonstrated that pembrolizumab and chemotherapy combination resulted in a significant improvement in PFS in patients with metastatic TNBC with ≥ CPS.^5^ Several studies indicated that HER2+ and HR+ BC patients observed the modest responses to PD‐L1 inhibitors. For HER2+ BC patients, phase 1 trial of PANACEA has shown 15% objective response rate in PD‐L1‐positive patients and no response in PD‐L1‐negative patients.^6^ Since HR+ tumors have less abundant tumor‐infiltrating lymphocyte levels and PD‐L1 level, the response rates are lower compared to TNBC and HER2+ BC patients.^7^, ^8^, ^9^ All the above reports highlight the importance of patient selection according to PD‐L1 immune cell expression.

Several gene mutations may affect the ability of tumor cells to evade immune surveillance. For example, TP53 mutations may serve as useful biomarkers for predicting response to cancer immunotherapy in different cancers.^10^ Mutation NOTCH4 acts as a biomarker related to a better response to ICI therapy,^11^ and PTEN mutation also may be a biomarker for ICIs in TNBC.^12^ The PD‐L1‐positive patients received atezolizumab in combination with nab‐paclitaxel achieved good efficacy regardless of *BRCA1/2* mutation status.^13^ Mutations in RYR2 and AHNAK were demonstrated to relate to favorable outcome in basal‐like breast tumors expressing PD‐L1.^14^ These findings raised that whether comprehensive genomic alterations in BC tissues are associated with the existed ICI biomarkers such as PD‐L1, and more specific genes can predict treatment outcome all need to be explored.

In this study, we sought to determine the genetic alteration profile and PD‐L1 level across different breast cancer subtypes in Chinese population, and investigate the correlations between them.

## 2 METHODS

### 2.1 Patient cohort

A total of 301 primary treatment‐naive BC patients in Chinese population including 16 ductal carcinoma in situ (DCIS), 251 infiltrative ductal cancer (IDC), and 34 other histological types were enrolled from Guangdong Provincial People's Hospital from December 2017 to July 2019. Their clinicopathologic parameters are listed in Table 1. Pathological characteristics, such as ER, PR, and HER2 levels in specimen were detected using immunohistochemistry (IHC) staining. The HR status was stratified into negative (HR−: ER− and PR−) and positive (HR+: ER+ and/or PR−). These BCs were classified into the HR+/HER2−, HR+/HER2+, HR−/HER2+, and HR−/HER2− subtypes according to the status of HR and HER2.^15^

**TABLE 1:** Baseline characteristics of cohort (*N* = 301)

Characteristics	*N* (%)
Age (median, range)	48 (23 ~ 83)
Young (<40)	68 (22.59%)
Old (≥40)	233 (77.41%)
Menopausal Status	
Premenopausal	168 (55.81%)
Postmenopausal	131 (43.52%)
Unknown	2 (0.66%)
TMB (median, range)	3.4 (0.5 ~ 53.8)
TMB‐H (≥ 10 muts/Mb)	23 (7.64%)
TMB‐L (< 10 muts/Mb)	278 (92.36%)
PD‐L1 group	
PD‐L1‐negative (CPS <1)	164 (54.49%)
PD‐L1‐positive (1 ≤ CPS <10)	99 (32.89%)
PD‐L1‐positive (CPS ≥10)	38 (12.62%)
Stage (%)	
I	69 (22.92%)
II	126 (41.86%)
III	40 (13.29%)
IV	26 (8.64%)
Unknown	40 (13.29%)
Histological grade	
I	12 (3.99%)
II	146 (48.50%)
III	99 (32.89%)
Unknown	44 (14.62%)
Histological type (%)	
DCIS	16 (5.32%)
IDC	251 (83.39%)
Other	34 (11.29%)
Ki67 (mean, range)	30% (1–100%))
Ki67‐H (≥20%)	226 (75.08%)
Ki67‐L (<20%)	75 (24.92%)
ER (mean, range)	90% (0–100%)
PR (mean, range)	20% (0–100%)
Molecular type	
HR+/HER2+	47 (15.62%)
HR+/HER2−	185 (61.46%)
HR−/HER2+	38 (12.62%)
TNBC	31 (10.30%)

### 2.2 Immunohistochemical staining

All resected specimens for IHC were formalin‐fixed, paraffin‐embedded (FFPE). Tumor subtypes are determined through obtaining ER, PR, HER2, and ki67 IHC staining. Staining for all markers was carried out according to the standard laboratory techniques. FFPE tissue sections for ER, PR, HER2, and Ki67 were stained, respectively. Ki67 ≥ 20% is defined as high (Ki67‐H) and Ki67 < 20% is defined as low (Ki67‐L). ER or PR expression were identified positive if >1% of tumor nuclei were strongly stained in accordance with the 2010 ASCO/CAP guidelines.^16^ Additionally, HER2 status was detected by fluorescence in situ hybridization.^17^

Immunohistochemical staining of FFPE tissues were performed by anti‐PD‐L1 antibodies (clone 22C3, Cat#M3653, DAKO). Dilutions (22C3 1:50) of the primary antibodies were used for antigen detection. All slides were counterstained with hematoxylin. PD‐L1 level was reported as CPS which was defined as the number of PD‐L1‐positive cells divided by the total tumor cells, multiplied by 100. CPS <1 is defined as PD‐L1‐negative; 1 ≤ CPS <10 is defined as PD‐L1‐low (PD‐L1‐L); CPS ≥10 is defined as PD‐L1‐high (PD‐L1‐H).

### 2.3 Genetic analysis

Targeted NGS of 450 cancer‐related genes was carried out in OrigiMed, Shanghai, China, for identification of genomic variants.^18^ Briefly, tumor specimen were formalin‐fixed, paraffin‐embedded, stained, and then pathologists believe that tumor tissue reaches 20% (tumor cell structure) mass before tumor cell content can be determined. Genomic DNA were isolated from FFPE samples and matched blood samples. Single nucleotide variants (SNVs), insertion‐deletions (Indels), copy number variation (CNV) regions, gene fusions, and gene rearrangements were analyzed using MuTect (v1.7), PINDEL (V0.2.5), control‐FREEC (v9.7), an in‐house developed pipeline, and integrative genomics viewer. The functional impact of GAs was annotated by SnpEff3.0.

TMB was calculated by counting somatic mutations.^18^ High TMB (TMB‐H) and low TMB (TMB‐L) were defined as ≥10 and < 10 mutations/megabase (muts/Mb), respectively.

### 2.4 Statistical analysis

SPSS software was carried out for statistical analyses. The relevance between PD‐L1 presence and genomic characteristics, the comparisons of PD‐L1 level between TMB‐high and ‐low patients, and the correlation between PD‐L1 and Ki67 expression were observed by Fisher's exact test. A value of *p* < 0.05 was recognized significant.

## 3 RESULTS

### 3.1 Patient characteristics

Clinical characteristics of 301 BC patients were observed in Table 1. The median patient age was 48 years (ranged from 23 to 83 years). The population was comprised of stage I, II, III, and IV patients, which accounted for 22.92%, 41.86%, 13.29%, and 8.64%, respectively. There were 137 (45.51%) BC patients, who were PD‐L1‐positive, of which 32.89% (1 ≤ CPS <10) were defined as PD‐L1‐L, while 12.62% (CPS ≥10) were defined as PD‐L1‐H. Ki67‐H was found in 75.08% of cases. The BC patients in our cohort were mainly grouped into the HR+/HER2−, HR+/HER2+, HR−/HER2+, and HR−/HER2− subtypes in accordance with the results of immunohistochemical staining, which accounted for 61.46%, 15.62%, 12.62%, and 10.30%, respectively.

### 3.2 Genetic alteration profile in Chinese patients with BCs

**FIGURE 1:**
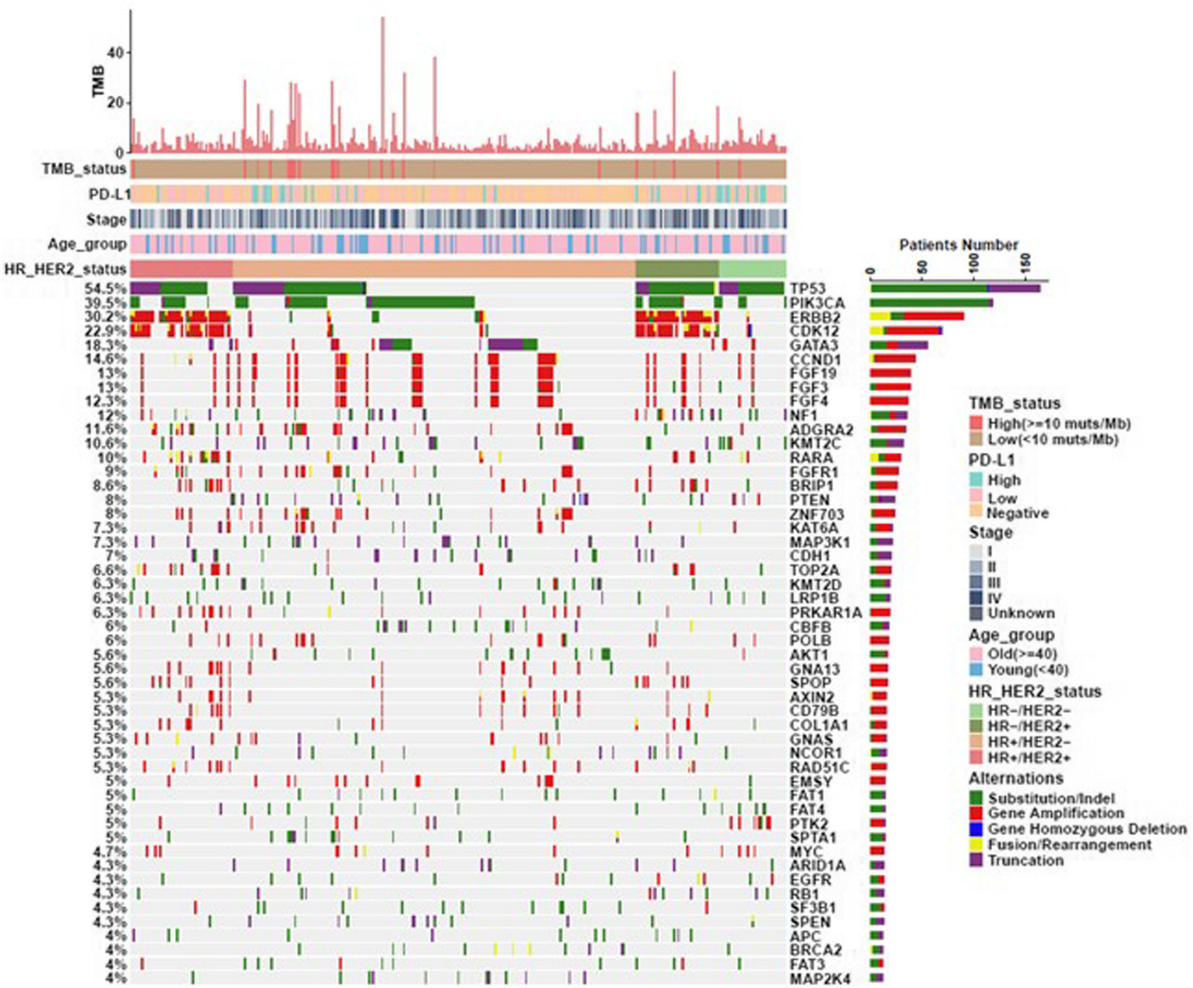
Mutational characteristics in BC. Genomic alteration profiles in BC.

There were 3,892 clinically relevant alterations identified from these 301 BC samples in Chinese population. TMB‐H was seen in 7.64% of cases while TMB‐L was 92.36%. Among all the alterations, 1781 (45.76%) were substitution/Indel, 1426 (36.64%) were gene amplification, 404 (10.38%) were truncation, 252 (6.47%) were fusion/rearrangement, and 29 (0.75%) were gene homozygous deletion. The most prevalent variations identified in our Chinese BC cohort was TP53 mutation (54.5%), followed by mutations in PIK3CA (39.5%), ERBB2 (30.2%), *CDK12* (22.9%), and *GATA3* (18.3%). The other top‐ranking mutated genes are shown in Figure 1.

**FIGURE 2:**
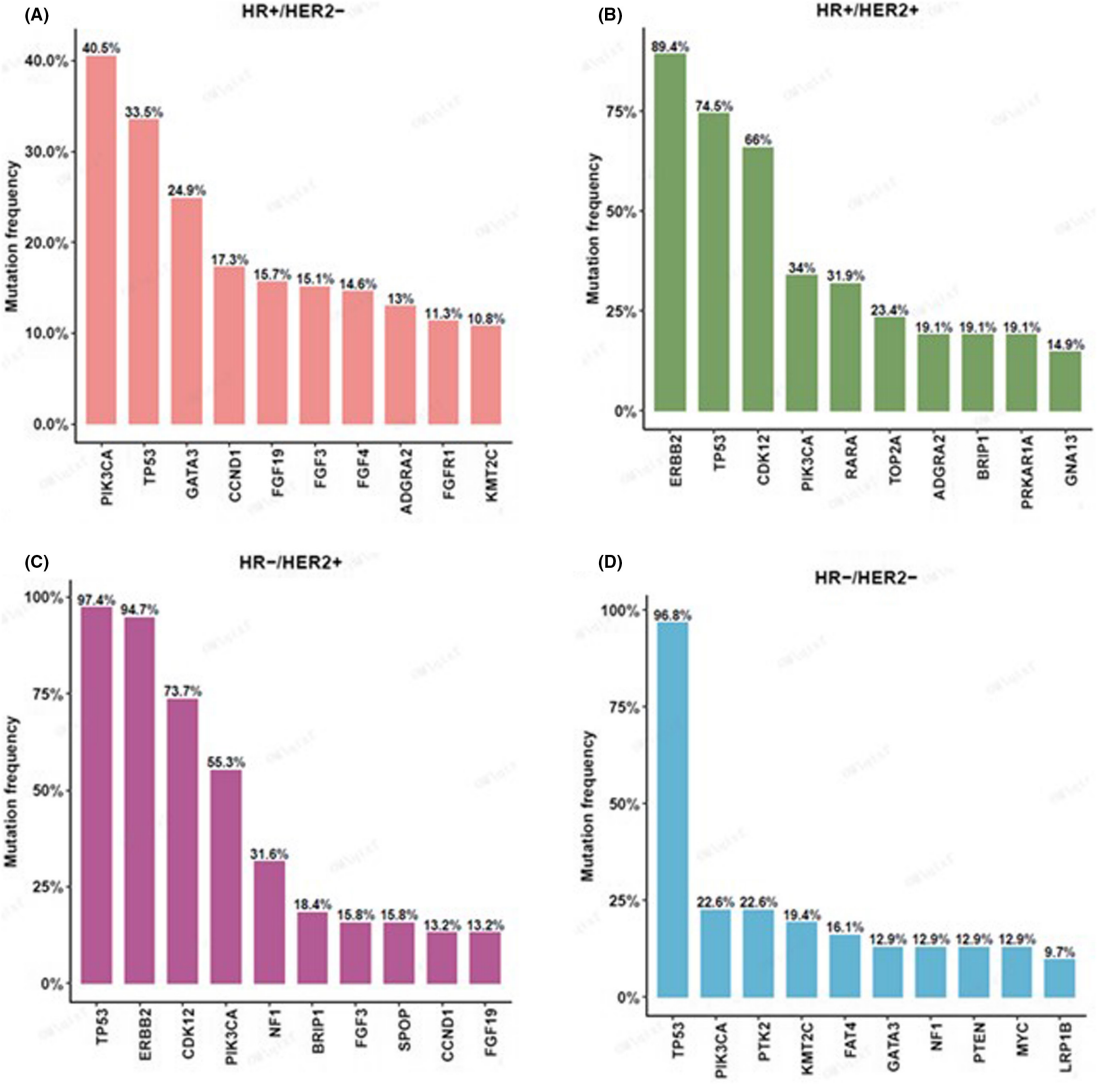
Mutational alterations were observed in different BC subtypes. The top ten frequency mutational alterations in (A) HR+/HER2−, (B) HR+/HER2+, (C) HR−/HER2+, and (D) TNBC subtypes.

**FIGURE 3:**
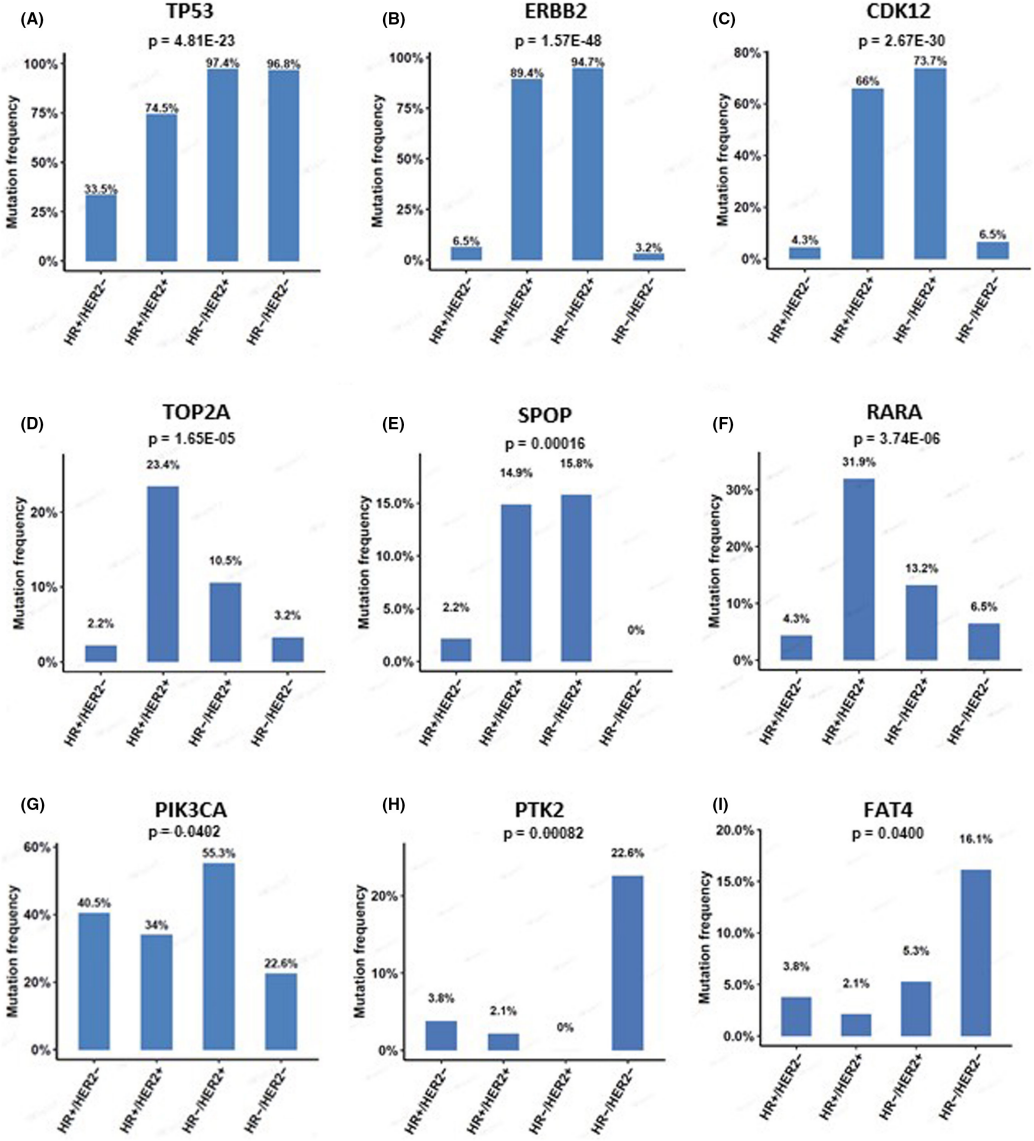
The mutated genes with statistical differences among BC subtypes. (A‐I) The mutation frequencies of TP53, ERBB2, CDK12, TOP2A, SPOP, RARA, PIK3CA, PTK2, and FAT4 are shown among BC subtypes. *p* < 0.05 is considered statistically significant.

Mutational profiles varied in different BC subtypes as shown in Figure S1. The most frequently altered genes were *PIK3CA* (40.5%), *TP53* (33.5%), *GATA3* (24.9%), *CCND1* (17.3%), and *FGF19* (15.7%) in HR+/HER2− subtype (Figure 2A), *ERBB2* (89.4%), *TP53* (74.5%), CDK12 (66%), *PIK3CA* (34%), and *RARA* (31.9%) in HR+/HER2+ subtype (Figure 2B), *TP53* (97.4%), *ERBB2* (94.7%), *CDK12* (73.7%), *PIK3CA* (55.3%), and *NF1* (31.6%) in HR−/HER2+ subtype (Figure 2C), and *TP53* (96.8%), *PIK3CA* (22.6%), *PTK2* (22.6%), *KMT2C* (19.4%), and *FAT4* (16.1%) in TNBC subtype (Figure 2D). The mutation frequencies of *TP53* (*p* = 4.81 E‐23), *ERBB2* (*p* = 1.57 E‐48) and *CDK12* (*p* = 2.67 E‐30), *TOP2A* (*p* = 1.65 E‐05), *SPOP* (*p* = 0.00016), RARA (*p* = 3.74 E‐06), PIK3CA (*p* = 0.0402), PTK2 (*p* = 0.00082), and FAT4 (*p* = 0.0400) were significantly different among the four BC subtypes (Figure 3), and so the other genes in these four subtypes in Table S1. Moreover, we found that several genes were mutated in individual subtypes (Table S1). *DDR2* (5.26%) and *MYCL* (5.26%) were only mutated in HR−/HER2+ subtype, and *H3‐3A* (9.68%), *NRAS* (6.45%) were associated with HR−/HER2− subtype. Differentially mutated genes in different subtypes may propose potential guiding significance for future therapeutic approaches.

### 3.3 Associations of PD‐L1 expression with clinical factors and genomic alterations

**FIGURE 4:**
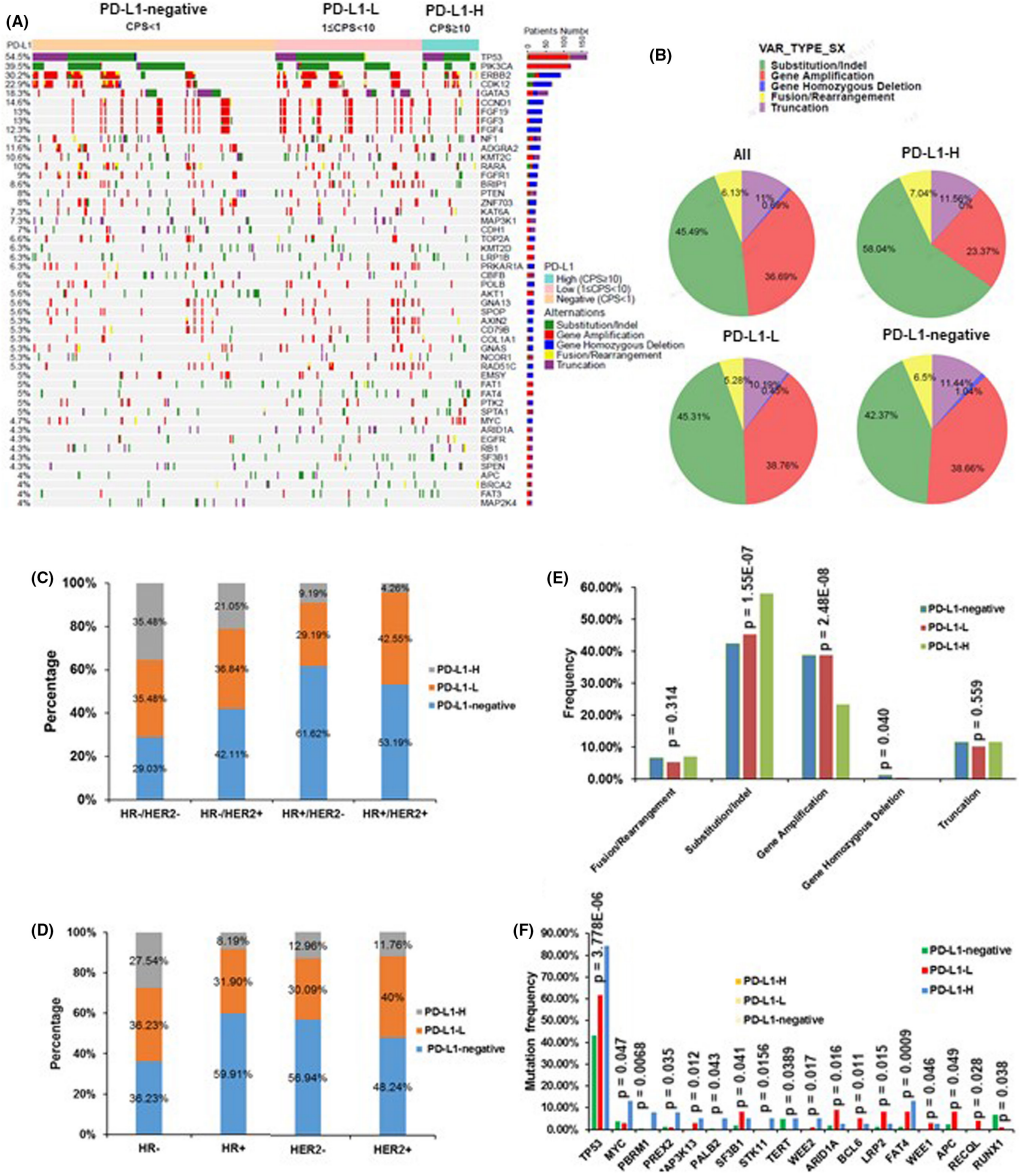
Heatmap comparing mutated genes according to different PD‐L1 levels. (A) The genomic alteration profiles is shown based on CPS. (B,E) The special alterations were observed in different BC subtypes. (C) The distributions of PD‐L1‐negative, PD‐L1‐H, and PD‐L1‐L in each subtypes of BC. (D) The distributions of PD‐L1‐negative, PD‐L1‐H, and PD‐L1‐L in HR−, HR+, HER2−, and HER2+ cohorts. (F) The statistical differences of mutational genes frequencies among groups based on PD‐L1 expression level. *p* < 0.05 is considered statistically significant.

Among the patients, 164 (54.5%) BCs were PD‐L1‐negative (CPS <1), while 137 (45.5%) BCs were PD‐L1‐positive (CPS ≥1) including 99 (72.3%) PD‐L1‐L and 38 PD‐L1‐H (27.7%) (Table 1). The distribution of patients with PD‐L1‐negative, PD‐L1‐H, and PD‐L1‐L in different BC subtypes were further analyzed. The percentage of patients with PD‐L1‐positive expression was higher in HR−/HER2− subgroup (70.9% vs. 38.4%, *p* = 0.0037) mainly due to the percentage of PD‐L1‐H level (35.5% vs. 9.2%, *p* = 0.0004). Patients in HR+/HER2− subgroup exhibited the lowest PD‐L1‐postive rate (38.4%), while patients in HR+/HER2+ showed the lowest percentage of PD‐L1‐H (4.26%) (Figure 4C). Moreover, we compared the PD‐L1 expressions among the whole HR+, HR−, HER2+, and HER2− subgroups (Figure 4D). Significantly higher PD‐L1‐H frequencies (27.5% vs. 8.2%, *p* = 0.0001) were observed in HR− patients compared to that in HR+ patients, while there was no difference between HER2− and HER2+ subgroups.

We also determined the relationship between PD‐L1 and clinicopathologic features, and observed that PD‐L1 level was remarkly related to ER, PR status and histological grade (Table 2). Mutational profiles of BCs with PD‐L1‐positive (CPS ≥1) and PD‐L1‐negative are shown in Figure 4A. The most frequent alteration type was substitution/Indel (45.49%), followed by gene amplification (36.69%), truncation (11.0%), fusion/rearrangement (6.13%), and gene homozygous deletion (0.69%; Figure 4B). The mutations have more substitutions/Indels (*p* = 1.55 E‐07), but less gene amplifications (*p* = 2.48 E‐08) in PD‐L1‐H BCs compared to that of the PD‐L1‐L and PD‐L1‐negative BCs (Figure 4E). Such differences were also observed for the mutation types and frequencies among PD‐L1‐H, PD‐L1‐L, and PD‐L1‐negative BC subsets. The top five genes in descending order were *TP53*, *PIK3CA*, *ERBB2*, *CDK12*, and *NF1* in PD‐L1‐H subset, and were *TP53*, *PIK3CA*, *ERBB2*, *CDK12*, and *GATA3* in both PD‐L1‐L and PD‐L1‐negative subsets (Figure S2). Moreover, the highest mutational frequencies of *TP53* (84.21% vs. 61.62% vs. 43.29%, *p* = 3.778 E‐06), *MYC* (13.16% vs. 3.03% vs. 3.66%, *p* = 0.047), *FAT4* (13.16% vs. 8.08% vs. 1.22%, *p* = 0.0009), *PBRM1* (7.89% vs. 0.0% vs. 0.61%, *p* = 0.007), and *PREX2* (7.89% vs. 1.01% vs. 1.22%, *p* = 0.035) were analyzed to be dramatically different in BC PD‐L1‐H cohort compared to that in PD‐L1‐L and PD‐L1‐negative cohort (Figure 4F).

**TABLE 2:** Comparison of clinical and molecular characteristics between PD‐L1 expression negative and positive

Characteristics	Negative	Positive		*p*
	PD‐L1 (CPS <1)	PD‐L1 (1 ≤ CPS < 10)	PD‐L1 (CPS ≥ 10)	
	*N* = 164	*N* = 99	*N* = 38	
Age				0.182
Young	35 (21.34%)	23 (23.23%)	13 (34.21%)	
Old	129 (78.66%)	76 (76.77%)	25 (65.79%)	
Menopausal status				0.632
Postmenopausal	74 (45.12%)	43 (43.43%)	14 (36.84%)	
Premenopausal	89 (54.27%)	55 (55.55%)	24 (63.16%)	
Unknown	1 (0.61%)	1 (1.01%)	0 (0.00%)	
ER Status				<0.000001
Negative	24 (14.63%)	20 (20.20%)	18 (47.37%)	
Positive	140 (85.37%)	79 (79.80%)	20 (52.63%)	
PR Status				0.000008
Negative	41 (25.00%)	37 (37.37%)	25 (65.79%)	
Positive	123 (75.00%)	62 (62.63%)	13 (34.21%)	
HER2 status				0.394
Negative	120 (73.17%)	65 (65.66%)	28 (73.68%)	
Positive	44 (26.83%)	34 (34.34%)	10 (26.32%)	
Stage				0.381
I	40 (24.39%)	21 (21.21%)	7 (18.42%)	
II	71 (43.29%)	35 (35.35%)	20 (52.63%)	
III	19 (11.58%)	18 (18.18%)	3 (7.89%)	
IV	16 (9.76%)	6 (6.06%)	4 (10.53%)	
Unknown	18 (10.98%)	19 (19.19%)	4 (0.00%)	
Histological grade				0.0005
I	12 (7.32%)	3 (3.03%)	0 (0.00%)	
II	89 (54.27%)	46 (46.46%)	9 (23.68%)	
III	41 (25.00%)	34 (34.34%)	24 (63.16%)	
Unknown	22 (13.41%)	16 (16.16%)	5 (13.16%)	

*Note*: CPS < 1 is defined as Low; 1 ≤ CPS < 10 is defined as Middle; CPS ≥10 is defined as High.

### 3.4 Correlation of PD‐L1 with Ki67 and TMB

**FIGURE 5:**
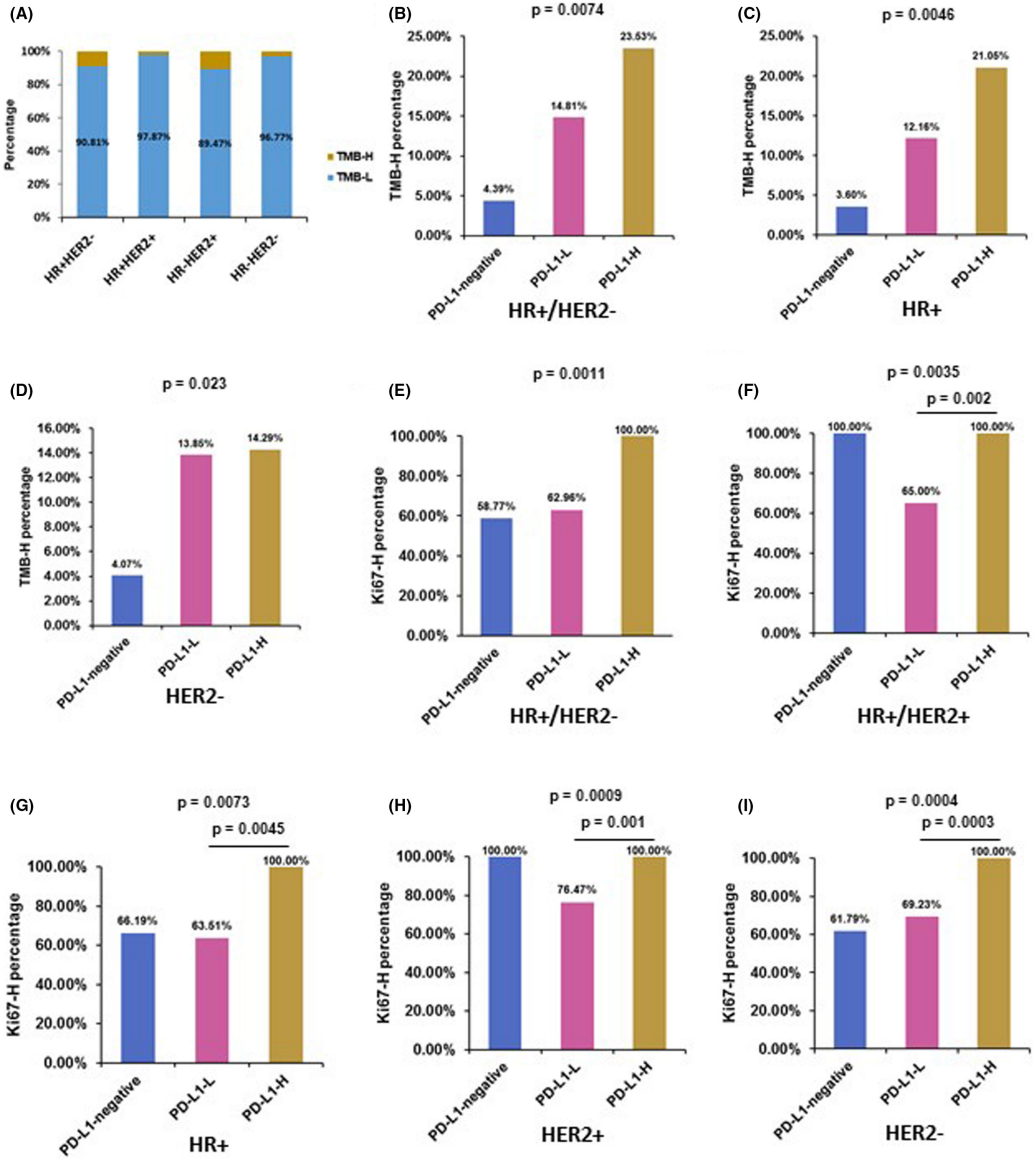
The correlation of TMB and Ki67 with PD‐L1 in different subtypes. The percentage of patients with high TMB was determined in (A) HR+/HER2− subtypes and (B) all BC patients with different CPS. The percentage of patients with high Ki67 was determined in (C) HR+/HER2− subtype, (D) HR+/HER2− subtype, and (E) all BC patients with different CPS. *p* < 0.05 is considered statistically significant.

TMB emerged recently as predictive factors to guide immunotherapies in real‐world clinical practice has attracted tremendous attention.^19^ First, we found that there was a little higher percentage of TMB‐H in HR + HER2− and HR−HER2+ subgroups (9.2% and 10.5%, respectively) than in HR−HER2− and HR + HER2+ subgroups (3.2% and 2.1%, respectively), though there was no statistical difference (Figure 5A). Then we analyzed the relationship between TMB and clinical factors, and demonstrated that postmenopausal status was obviously related to TMB‐H (*p* = 0.018, Table 3). We also showed a positive correlation between TMB and PD‐L1 in HR+/HER2− cohort (*p* = 0.0074), HR+ cohort (p = 0.0046), HER2− cohort (p = 0.023), and all BCs (*p* = 0.007) cohort (Figure 5B‐D, Figure S3A and Table S2), indicating a higher percentage of TMB‐H BCs in the PD‐L1‐positive BCs than in the PD‐L1‐negative BCs. In HER2− cohort, there were more PD‐L1‐H than PD‐L1‐L patients (*p* = 0.032).

**TABLE 3:** Comparison of clinical and molecular characteristics between TMB‐high and TMB‐low

Characteristics	TMB‐H	TMB‐L	*p*
	*N* = 23	*N* = 278	
Age			0.12
Young	2 (8.70%)	66 (23.74%)	
Old	21 (91.30%)	212 (76.26%)	
Menopausal status			0.018
Postmenopausal	16 (69.57%)	115 (41.36%)	
Premenopausal	7 (30.43%)	161 (57.91%)	
Unknown	0 (0.00%)	2 (0.72%)	
ER Status			0.898
Negative	5 (21.74%)	57 (20.50%)	
Positive	18 (78.26%)	221 (79.50%)	
PR status			0.773
Negative	9 (39.13%)	94 (33.81%)	
Positive	14 (60.87%)	184 (66.19%)	
HER2 status			0.559
Negative	18 (78.26%)	195 (70.14%)	
Positive	5 (21.74%)	83 (29.86%)	
Stage (%)			0.746
I	4 (17.39%)	64 (23.02%)	
II	6 (26.09%)	120 (43.17%)	
III	3 (13.04%)	37 (13.31%)	
IV	2 (8.70%)	24 (8.63%)	
Unknown	8 (34.78%)	33 (11.87%)	
Histological grade (%)			0.547
I	0 (0.00%)	15 (5.40%)	
II	12 (52.17%)	132 (47.48%)	
III	10 (43.48%)	89 (32.01%)	
Unknown	1 (4.35%)	42 (15.11%)	

A high Ki67 index (approximately ≥20%) is related to malignant phenotype, poor prognosis in BC.^20^ We demonstrated that high Ki67 index was negatively correlated with ER (*p* = 1.25 E‐26) and PR status (*p* = 3.36 E‐07), and was positively related to HER2 status (*p* = 0.0008) and histological grade (*p* = 0.0005, Table 4). Moreover, the proportion of patients with Ki67‐H was higher in patients with PD‐L1‐H in HR+/HER2− cohort (*p* = 0.001), HR+/HER2− cohort (*p* = 0.002), HR+ cohort (*p* = 0.004), HER2+ cohort (*p* = 0.001), HER2− cohort (*p* = 0.0003), and all BC patients (*p* = 0.0006), but were not associated with TMB levels (Figure 5E‐I, Figure S3B, Tables S3 and S4). These results may reveal more possibilities for the treatment of BC with different subtypes.

**TABLE 4:** Comparison of clinical and molecular characteristics between Ki67‐high and Ki67‐low

Characteristics	Ki67‐H	Ki67‐L	*p*
	*N* = 226	*N* = 75	
Age			0.157
Young	56 (24.78%)	12 (16.00%)	
Old	170 (75.22%)	63 (84.00%)	
Menopausal status			1
Postmenopausal	99 (43.80%)	32 (42.67%)	
Premenopausal	126 (55.75%)	42 (56.00%)	
Unknown	1 (0.44%)	1 (1.33%)	
ER status			1.25 E‐26
Negative	165 (73.01%)	1 (1.33%)	
Positive	61 (26.99%)	74 (98.67%)	
PR status			3.36 E‐07
Negative	96 (42.48%)	7 (9.33%)	
Positive	130 (57.52%)	68 (90.67%)	
HER2 status			0.0008
Negative	148 (65.49%)	65 (86.67%)	
Positive	78 (34.51%)	10 (13.33%)	
Stage (%)			0.518
I	51 (22.57%)	18 (24.00%)	
II	94 (41.59%)	32 (42.67%)	
III	33 (14.60%)	7 (9.33%)	
IV	22 (9.73%)	4 (5.33%)	
Unknown	26 (11.50%)	14 (18.67%)	
Histological grade (%)			0.0005
I	5 (2.21%)	10 (13.33%)	
II	101 (44.69%)	43 (57.33%)	
III	92 (40.71%)	7 (9.33%)	
Unknown	28 (12.38%)	15 (20.00%)	

## 4 DISCUSSION

In our study, we systematically analyzed the association of the genome with the PD‐L1 to determine the relationship between genetic mutations and biomarkers for ICI. Our study explored the mutational alterations based on the PD‐L1 levels, and determined the specific mutated genes in the different BC subtypes. Additionally, we observed the association with PD‐L1 and Ki67 or TMB, helping introduce more connections of ICIs with other factors.

BC is a heterogeneous disease, and clinical outcome and response to therapy vary by subtype. Different BC subtypes can be identified by different biomarkers with different clinical characteristics that will influence the treatment of patients.^21^ The Cancer Genome Atlas Network observed the HR+/HER2− subtype harbored the most frequent mutations being PIK3CA, MAP3K1, and TP53; HER2+ subtype had a high frequency of TP53 and PIK3CA, and basal‐like cancers had a high frequency of TP53 mutations.^21^ In our Chinese cohort, the most frequency mutations of *PIK3CA*, *TP53*, and *GATA3* occurred in HR+/HER2− subtype; Mutations *ERBB2*, *TP53*, and CDK12 were observed in HR+/HER2+ subtype; *TP53*, *ERBB2,* and *CDK12* mutations occurred in HR−/HER2+ subtype; and *TP53*, *PIK3CA,* and *PTK2* mutations frequently occurred in TNBC subtype. Most notably, the mutation frequencies of *TP53*, *ERBB2* and *CDK12*, *TOP2A*, *SPOP*, *RARA*, *PIK3CA*, *PTK2*, and *FAT4* were significantly different among the four BC subtypes. Meric–Bernstam et al. have shown that mutation TP53 was related to a shorter OS in HR+ BCs, and were not related to survival in TNBC or HER2+ BCs.^22^ Our study observed TP53 mutations were significantly higher in HR−/HER2+ and TNBC subtypes, which might represent a poor prognosis for patients with this subtype. This is also a limitation of our study, the prognostic data for the patients were not collected. One study was designed to assess the efficacy of the irreversible pan‐HER kinase inhibitor, neratinib, alone or in combination with fulvestrant, in metastatic BC patients with ERBB2 mutations.^23^ The frequency of ERBB2 and CDK12 mutations over 66% occurred in HR−/HER2+ and HR+/HER2+ subtypes, which was much higher than in the other two subtypes. In BC, the RARA gene is mutated and amplified, and the gene interacts with the ER.^24^ Metastatic castration‐resistant prostate cancer patients with SPOP somatic mutations have higher response rates and treatment durations to the novel endocrine therapy abiraterone^25^; In our study, SPOP was remarkly higher in HR−/HER2+ subtype compared to that in HR+/HER2− and TNBC subtypes, and PTK2 and FAT4 were obviously higher in TNBC subtype than in the other three subtypes. The variation in the frequency of mutations among these subtypes may provide information for treatment and immunodrug use among the subtypes. *PRKAR1A* mutations were highly mutated in HR+/HER2+ subtype and *DDR2* was only mutated in HR−/HER2+ subtype. A BC patient harbored a rare *PRKAR1A* R228 mutation and obtained appropriate targeted therapy.^26^ In the near future, molecular testing will help to better understand disease biology and tailor therapeutic strategies for each specific individual.

Immunotherapy is one of the most encouraging finding of cancer therapy in recent years. BC features that might be related to the response to ICIs include a higher mutational load, higher PD‐L1 level, and enhanced TILs.^27^, ^28^, ^29^ Immunomodulation/immunotherapy (either PD1 or PD‐L1 antibody) is a good choice for the patient with PD‐L1‐positive status, and PD‐L1 has been reported as an active immune checkpoint in a variety of cancers including BC. Atezolizumab has been approved as the first ICB agents for TNBC patients with PD‐L1 expression.^2^ The IMPASSION 130 study has shown that improved PFS in PD‐L1‐positive TNBC subgroup treating with atezolizumab plus nab‐paclitaxel.^4^ HER2+ and HR+ BC patients indicated modest responses to PD‐L1 inhibitors. For HER2+ BC, phase 1 trial of PANACEA showed a 15% ORR in PD‐L1‐positive patients and no responses among PD‐L1‐negative patients.^6^ In our study, genomic alterations were compared between PD‐L1‐positive and PD‐L1‐negative patients. The significantly higher mutational frequencies of *TP53*, *MYC*, *FAT4*, *PBRM1*, *PREX2*were observed in BC PD‐L1‐H cohort compared with that in PD‐L1‐L and PD‐L1‐negative cohort. The PD‐L1‐positive patients with these mutational genes might be more effective for immunotherapy or receiving a better prognosis.

There was some limitations of PD‐L1 as a biomarker because of its dynamic and heterogeneous expression in the tumor microenvironment, variable assay interpretation, and lack of standardization between platforms,^30^ tumor types.^31^, ^32^, ^33^, ^34^, ^35^ Higher TMB was observed in metastatic BC than that in primary cancers, and was shown associated with HR− BC and HER2+ BC.^27^ However, the predictive value of TMB for BC immunotherapy in BC is still controversial.^28^ Several studies observed that PD‐L1 and TMB act as independent predictors of response against ICB and the correlation between PD‐L1 level and TMB is low in multiple tumor types.^36^ While the landmark IMpassion130 trial revealed that TMB predicted better benefit to ICB in PD‐L1‐positive patients.^4^ There were limitations of PD‐L1 as a biomarker, thus establishing the independent benefit of TMB in predicting response to anti‐PD‐1 /PD‐L1 treatment as a very useful clinical tool. Furthermore, we observed a positive correlation between TMB and PD‐L1 in HR+/HER2− subtype, and showed that the proportion of patients with Ki67‐H was higher in patients with PD‐L1‐H in HR+/HER2− and HR+/HER2+ cohorts, but were not associated with TMB levels. The relationship among the predictive values of PD‐L1, TMB, and Ki67 may provide more possibilities for the treatment of BC with different subtypes. Importantly, more clinical trials need to be designed to examine clinical outcomes based on PD‐L1 level, Ki67, and/or TMB combinations that, when combined, can identify different populations that cannot be identified when used alone.

## 5 CONCLUSION

We analyzed the mutational alterations in BC patients with HR+, TNBC, and HER2+ subtypes, and determined the special alterations among different subtypes. In addition, we explored the mutational alterations based on PD‐L1 levels, and determined the specific mutated genes in the different BC subtype. Moreover, we analyzed the association with TMB and Ki67, helping introduce new therapies to treat specific patient subgroups. Molecular testing will allow better understanding of disease biology and tailor therapy for specific individual. Verification of using integrated predictive biomarkers for selecting checkpoint inhibitors as a component of combination systemic therapy in BC is ongoing.

## AUTHOR CONTRIBUTIONS

Kai Li, Li Cao, Cheukfai Li, Jundong Wu, Bo Chen Xiaomin Liang, and Zhaoyang Wang were involved in conceptualization, data curation. Guochun Zhang, Xueri Li, Lingzhu Wen, Minghan Jia, Guangnan Wei, Jiali Lin Yifan Zhao, Wen Xiao, Lin Du, and Meng Wang participated in formal analysis, investigation. Yingzi Li, Yuchen Zhang, Hsiaopei Mok, Chongyang Ren, Yulei Wang were involved in data curation. Xiaofang Qi, Lijie Guo, and Yue Che were involved in data curation, formal analysis, investigation, methodology, software, and visualization. Kai Li, Li Cao, Cheukfai Li, and Ning Liao participated in conceptualization, funding acquisition, project administration, resources, supervision, validation, writing, reviewing, and editing the original draft.

## DECLARATION OF COMPETING INTEREST

The authors have no conflict of interest to declare.

## FUNDING INFORMATION

This study was supported by funding from Supporting start‐up funds of Guangdong Provincial People's Hospital for National Natural Science Foundation of China (KY012019326; 8190100873; K19010101).

## ETHICS STATEMENT

Primary tumor biopsies were obtained using an Institutional Review Board approved protocol, and this study had been approved by the Ethics Committee of Guangdong Provincial People's Hospital (No. GDREC2014122H). All patients provided written informed consent for translational research. We thank all the patients and their families for participation. Informed consent was obtained from all the individual participants included in the study.

## Supporting information


Figure S1.

Figure S2.

Figure S3.

Table S1.
Table S2.
Table S3.
Table S4.

## Data Availability

Some or all data, models, or code that support the findings of this study are available from the corresponding author upon reasonable request
