# Supplementary figures and images for: Genomic alteration profile and PD‐L1 expression among different breast cancer subtypes in Chinese population and their correlations
Source: Cancer Med. 2022 Nov 20;12(5):5195–208. doi: 10.1002/cam4.5314 (PMC10028068; doi:10.1002/cam4.5314)

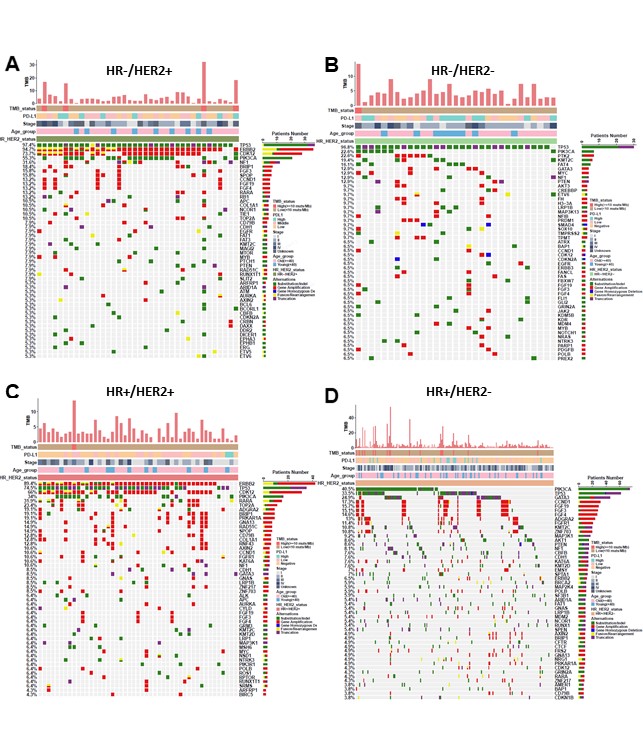

Supplement: Supplementary file 1 — Figure S1. [file CAM4-12-5195-s001.jpg]

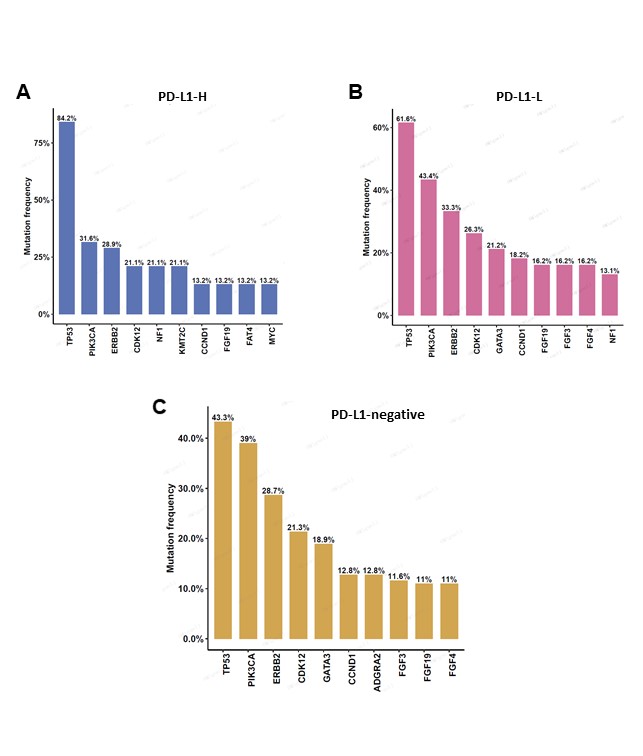

Supplement: Supplementary file 2 — Figure S2. [file CAM4-12-5195-s002.jpg]

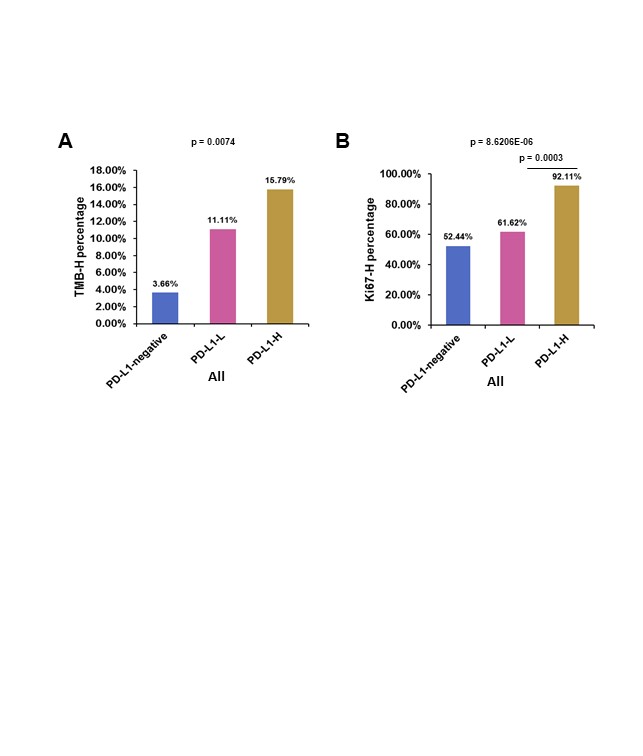

Supplement: Supplementary file 3 — Figure S3. [file CAM4-12-5195-s003.jpg]
