# Supplementary material for: Genomic alteration profile and PD‐L1 expression among different breast cancer subtypes in Chinese population and their correlations
Source: Cancer Med. 2022 Nov 20;12(5):5195–208. doi: 10.1002/cam4.5314 (PMC10028068; doi:10.1002/cam4.5314)
Supplement: Supplementary file 4 — Table S1. Table S2. Table S3. Table S4. [file CAM4-12-5195-s004.docx]

Supplementary Table 1. Different mutational genes in four subtypes.

| **Gene** | **HR-/HER2-** | **HR+/HER2+** | **HR+/HER2-** | **HR-/HER2+** | **p** |
| --- | --- | --- | --- | --- | --- |
| ADGRA2 | 0.0323 | 0.1915 | 0.1297 | 0.0263 | 0.041949027 |
| AKT1 | 0.0323 | 0 | 0.0865 | 0 | 0.032366688 |
| AKT3 | 0.0968 | 0.0213 | 0.0108 | 0 | 0.040320962 |
| BRIP1 | 0.0323 | 0.1915 | 0.0486 | 0.1842 | 0.001442561 |
| CAMTA1 | 0.0323 | 0.0426 | 0 | 0 | 0.034892832 |
| CD79B | 0 | 0.1277 | 0.0378 | 0.0789 | 0.038850425 |
| COL1A1 | 0.0323 | 0.1277 | 0.027 | 0.1053 | 0.014681497 |
| CRBN | 0.0323 | 0 | 0 | 0.0526 | 0.015246614 |
| DDR2 | 0 | 0 | 0 | 0.0526 | 0.025869324 |
| EPHA3 | 0.0323 | 0.0426 | 0.0054 | 0.0526 | 0.044757289 |
| EPHA5 | 0.0323 | 0.0426 | 0 | 0 | 0.034892832 |
| ERG | 0 | 0.0213 | 0 | 0.0526 | 0.027445861 |
| ETV6 | 0.0968 | 0.0213 | 0 | 0.0526 | 0.001258548 |
| FH | 0.0968 | 0.0213 | 0.0054 | 0.0526 | 0.008101935 |
| GATA3 | 0.129 | 0.0851 | 0.2486 | 0.0263 | 0.000856547 |
| GNA13 | 0 | 0.1489 | 0.0486 | 0.0263 | 0.030614521 |
| H3-3A | 0.0968 | 0 | 0 | 0 | 0.0009989 |
| IGF1R | 0.0323 | 0.0426 | 0 | 0.0263 | 0.023838654 |
| KDM5C | 0.0323 | 0 | 0 | 0.0526 | 0.015246614 |
| MAP3K13 | 0.0968 | 0.0213 | 0.0054 | 0 | 0.014238876 |
| MTOR | 0.0323 | 0 | 0.0108 | 0.0789 | 0.049288006 |
| MYB | 0.0645 | 0.0213 | 0.0108 | 0.0789 | 0.026458375 |
| MYCL | 0 | 0 | 0 | 0.0526 | 0.025869324 |
| NF1 | 0.129 | 0.1064 | 0.0811 | 0.3158 | 0.002353029 |
| NFIB | 0.0968 | 0 | 0.0054 | 0 | 0.01698492 |
| NRAS | 0.0645 | 0 | 0 | 0 | 0.010299003 |
| NTRK3 | 0.0645 | 0.0638 | 0.0108 | 0.0526 | 0.036736178 |
| PDGFB | 0.0645 | 0.0213 | 0 | 0.0526 | 0.005930889 |
| PRKAR1A | 0 | 0.1915 | 0.0486 | 0.0263 | 0.003194835 |
| QKI | 0.0645 | 0 | 0 | 0 | 0.010299003 |
| RAD51C | 0 | 0.1489 | 0.0324 | 0.0789 | 0.008784291 |
| RAD52 | 0.0645 | 0 | 0 | 0.0263 | 0.010403671 |
| RB1 | 0.0645 | 0.0213 | 0.027 | 0.1316 | 0.029566921 |
| RNF43 | 0 | 0.1277 | 0.027 | 0 | 0.012444621 |
| RUNX1T1 | 0.0645 | 0.0638 | 0.0162 | 0.0789 | 0.046285541 |
| SMAD4 | 0.0968 | 0.0213 | 0 | 0 | 0.001985764 |
| SOX10 | 0.0968 | 0.0213 | 0 | 0 | 0.001985764 |
| TIE1 | 0 | 0 | 0.0162 | 0.1053 | 0.024310037 |
| TMPRSS2 | 0.0968 | 0 | 0.0108 | 0 | 0.027230579 |
| TPMT | 0.0968 | 0 | 0 | 0 | 0.0009989 |
| VEGFA | 0.0645 | 0 | 0.0054 | 0.0263 | 0.047192784 |
| ZNF703 | 0 | 0.0851 | 0.1081 | 0 | 0.030977666 |

Supplementary Table 2. Correlation analysis of PD-L1 and TMB.

|  | **Negative** |  | **Positive** | |  | |
| --- | --- | --- | --- | --- | --- | --- |
| **Subtypes** | **(CPS < 1)** |  | **(1 ≤ CPS < 10)** | **(CPS ≥ 10)** | | **p** |
|  | N = 164 |  | N = 99 | N = 38 | |  |
| HR+/HER2+ |  |  |  |  | | 0.468 |
| TMB-H | 0 (0.00%) |  | 1 (1.01%) | 0 (0.00%) | |  |
| TMB-L | 25 (15.24%) |  | 19 (19.19%) | 2 (5.26%) | |  |
| HR+/HER2- | |  |  |  | | 0.007 |
| TMB-H | 5 (3.05%) |  | 8 (8.08%) | 4 (10.53%) | |  |
| TMB-L | 109 (66.46%) |  | 46 (46.46%) | 13 (34.21%) | |  |
| HR-/HER2+ | |  |  |  | | 0.406 |
| TMB-H | 1 (0.61%) |  | 1 (1.01%) | 2 (5.26%) | |  |
| TMB-L | 15 (9.15%) |  | 13 (13.13%) | 6 (15.79%) | |  |
| HR-/HER2- |  |  |  |  | | 1 |
| TMB-H | 0 (0.00%) |  | 1 (1.01%) | 0 (0.00%) | |  |
| TMB-L | 9 (5.49%) |  | 10 (10.10%) | 11 (28.95%) | |  |

CPS **<** 1 is defined as Low; 1 **≤** CPS **<** 10 is defined as Middle; CPS ≥ 10 is defined as High.

Supplementary Table 3. Correlation analysis of PD-L1 and Ki67.

|  | **Negative** |  | **Positive** | |  | |
| --- | --- | --- | --- | --- | --- | --- |
| **Subtypes** | **(CPS < 1)** |  | **(1 ≤ CPS < 10)** | **(CPS ≥ 10)** | | **p** |
|  | N = 164 |  | N = 99 | N = 38 | |  |
| HR+/HER2+ |  |  |  |  | | 0.004 |
| Ki67-H | 25 (15.24%) |  | 13 (13.13%) | 2 (5.26%) | |  |
| Ki67-L | 0 (0.00%) |  | 7 (7.07%) | 0 (0.00%) | |  |
| HR+/HER2- | |  |  |  | | 0.001 |
| Ki67-H | 67 (40.85%) |  | 34 (34.34%) | 17 (44.74%) | |  |
| Ki67-L | 47 (28.66%) |  | 20 (20.20%) | 0 (0.00%) | |  |
| HR-/HER2+ | |  |  |  | | 0.579 |
| Ki67-H | 16 (9.76%) |  | 13 (13.13%) | 8 (21.05%) | |  |
| Ki67-L | 0 (0.00%) |  | 1 (1.01%) | 0 (0.00%) | |  |
| HR-/HER2- |  |  |  |  | | 1 |
| Ki67-H | 9 (5.49%) |  | 11 (11.11%) | 11 (28.95%) | |  |
| Ki67-L | 0 (0.00%) |  | 0 (0.00%) | 0 (0.00%) | |  |

CPS **<** 1 is defined as Low; 1 **≤** CPS **<** 10 is defined as Middle; CPS **≥** 10 is defined as High.

Supplementary Table 4. Correlation analysis of TMB and Ki67.

|  | **TMB-H TMB-L** | | | **p** |
| --- | --- | --- | --- | --- |
| **Subtypes** | **(≥ 10 muts/Mb)** | | **(< 10 muts/Mb)** |  |
|  | N = 23 | | N = 278 |  |
| HR+/HER2+ |  | |  | 1 |
| Ki67-H | 1 (4.35%) | | 39 (14.03%) |  |
| Ki67-L | 0 (0.00%) | | 7 (2.52%) |  |
| HR+/HER2- | |  | 0.159 | |
| Ki67-H | 14 (60.87%) | | 104 (37.41%) |  |
| Ki67-L | 3 (13.04%) | | 64 (23.02%) |  |
| HR-/HER2+ | |  | 1 | |
| Ki67-H | 4 (17.39%) | | 33 (11.87%) |  |
| Ki67-L | 0 (0.00%) | | 1 (0.36%) |  |
| HR-/HER2- |  | |  | 1 |
| Ki67-H | 1 (4.35%) | | 30 (10.79%) |  |
| Ki67-L | 0 (0.00%) | | 0 (0.00%) |  |
